# Population Dynamics of Bacterial Persistence

**DOI:** 10.1371/journal.pone.0062814

**Published:** 2013-05-13

**Authors:** Pintu Patra, Stefan Klumpp

**Affiliations:** Max Planck Institute of Colloids and Interfaces, Science Park Golm, Potsdam, Germany; Tel Aviv University, Israel

## Abstract

Persistence is a prime example of phenotypic heterogeneity, where a microbial population splits into two distinct subpopulations with different growth and survival properties as a result of reversible phenotype switching. Specifically, persister cells grow more slowly than normal cells under unstressed growth conditions, but survive longer under stress conditions such as the treatment with bactericidal antibiotics. We analyze the population dynamics of such a population for several typical experimental scenarios, namely a constant environment, shifts between growth and stress conditions, and periodically switching environments. We use an approximation scheme that allows us to map the dynamics to a logistic equation for the subpopulation ratio and derive explicit analytical expressions for observable quantities that can be used to extract underlying dynamic parameters from experimental data. Our results provide a theoretical underpinning for the study of phenotypic switching, in particular for organisms where detailed mechanistic knowledge is scarce.

## Introduction

**Figure 1:**
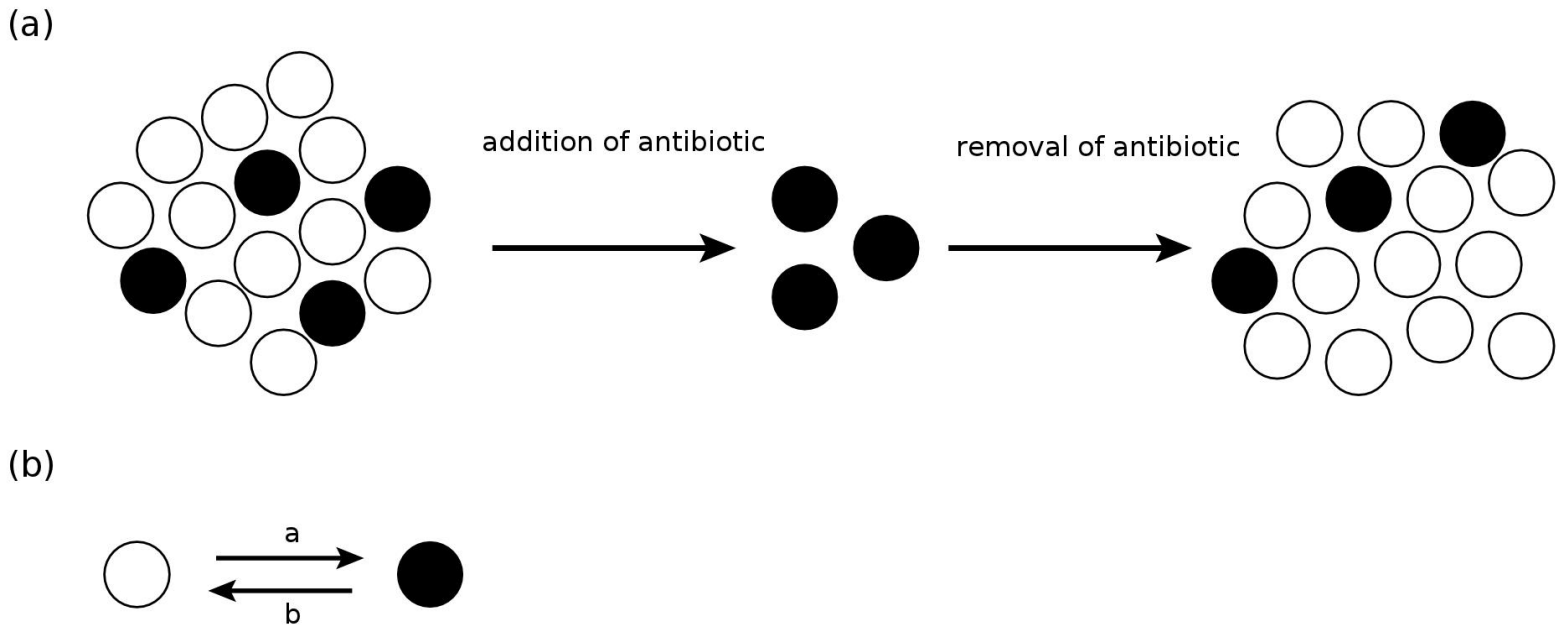
Phenotypic heterogeneity in a bacterial population. (a) Dynamics of heterogeneous population consisting of normal (white) and persister (black) cells: The persisters survive the addition of an antibiotic, and allow the population to grow back after the removal of the antibiotic. (b) Phenotype type switching: Cells stochastically switch between the normal and persister state with rates 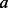 and 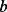.

The life of microorganisms is characterized by two main tasks, rapid growth and proliferation under conditions permitting growth and survival under stressful conditions [1]. One strategy to cope with such varying environmental conditions is phenotypic heterogeneity, the splitting of a genetically homogeneous population into subpopulations that execute different strategies for survival [2]–[4]. Phenotypic tolerance to antibiotics (persistence) is a prime example of such phenotypic heterogeneity: When a bacterial culture is treated with an antibiotic, typically a small fraction of the population, the persisters, survives and allows the culture to grow back once the antibiotic has been removed (Fig. 1), thus making it difficult to eradicate the population [5]–[7]. The re-grown culture remains susceptible to the antibiotic with the exception of yet again a small fraction of persisters, indicating that, in contrast to resistance, persistence is a phenotypic effect. Indeed observations at a single cell level have shown that cell switch in a stochastic fashion between the persister state and the normal state [8]. Moreover these experiments have shown that persistence is not an adaptive response to the antibiotics, but rather that persisters are present in the population before the antibiotic treatment [8] (there is however evidence that adaptive responses also play a role in some situations [9], [10]). The persister cells present in the population before treatment were shown to grow much more slowly than normal cells [8], [11], indicating that persistence while providing a fitness benefit (survival advantage) under stress conditions also invokes a fitness cost under unstressed conditions. Persistence is thus based on the coexistence of subpopulations growing with different growth rates. Mechanistically, the formation of persisters has been linked to the expression of chromosomal toxin-antitoxin systems [12]–[15], which are believed to give rise to a genetic circuit that exhibits bistable behavior resulting in subpopulations with different phenotypes characterized by different growth rates [16]–[19]. Indeed, experimental and theoretical studies of the coupling of gene expression and cell growth indicate that such growth bistability should be considered a rather generic phenomenon that can arise when gene circuits modulate cell growth [17], [20].

The molecular mechanisms for the generation of persisters are currently a topic of very active research. Persistence has been observed in a wide range of bacterial species [6], [7], [21], but on the mechanistic level, so far relatively little is known for bacteria other than the model organism *E. coli*. In the absence of detailed mechanistic knowledge, the main window into persistence is the study of the population dynamics upon antibiotic treatment, in particular, the survival upon administration of the drug and the re-growth of the population upon removal of the drug. Here we study a theoretical model for this dynamics that was originally proposed by Balaban et al. [8]. We make use of an excellent approximation (based on the assumption that the rates of phenotype switching are small, which is typically the case) to derive explicit analytical expression for a number of observable quantities for several typical experimental scenarios: constant environment, shift from growth to stress conditions or vice versa and periodically switching environments. Our analysis is similar to previous theoretical studies on phenotype switching [22], [23]. A small but important difference to the systematic perturbative approach used in Ref. [22] is that our theory is based on the approximation of small phenotype switching rates (as compared to the growth and death rates), while the approximation of Ref. [22] is based on long durations of environmental durations, such that populations structures reach their steady state before the environment changes. The latter is not required in our approximation and our approach thus allows us to study both short and long environmental durations (while long durations are expected to be typical for the natural environment, and thus appropriate for an evolutionary comparison of different modes of phenotype switching, such as stochastic and adaptive [22], short durations may be of importance for some experimental situations, such as resuscitation experiments after short periods of antibiotic treatment). We also note that while the mathematics of ours and the previous study are closely related, the scope of the studies is different. Rather than aiming at a general theoretical framework for phenotype switching phenomena, our goal here is to obtain simple explicit expression for measurable quantities. These expressions can be used to analyze experimental data for population growth and decay to provide insights into the mechanism of persistence based on simple population-scale experiments. The key results that may be used for the analysis of experimental data are summarized in a section 'Overview of key results' directly after the description of the model, while the remaining sections report the systematic analysis of the population dynamics.

## Model

We consider a bacterial population where an individual cell can have two distinct phenotypic states (Fig. 1) which are characterized by different sensitivities to given environmental conditions. The environmental sensitivity is reflected in growth and decay rates of the subpopulation in the given environment. For instance, in the case of persister cell on which we focus, normal cells are more sensitive to various stresses, i.e. they decay faster under various stress conditions such as antibiotic treatment [8] and phage attack [24], but also grow faster in unstressed conditions.

A cell in the normal state can switch to the persister state with rate 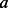 and a cell in the persister state can switch back to the normal state with rate 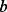. The instantaneous switching between phenotypic states leads to distinct subpopulation of normal and persister cells which compose the total population. We denote the growth rate of normal cells (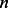) and persister cells (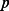) by 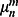 and 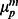 respectively, where 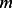 indicates the growth medium or, more generally the growth conditions. Below we will use indices '
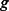
' and '
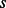
' to denote unstressed growth and stress conditions, respectively (e.g., growth medium not containing or containing an antibiotic). The resulting population dynamics can be described by the following system of equations [8], 
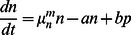


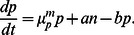
(1)


These equations are linear and their exact solution can be obtained [8]. Below we will obtain approximative solutions based on the assumption that the switching rates are small compared to the growth rates, which allows us to derive relatively simple explicit expressions for observable quantities.

## Results and Discussion

### Overview of key results

Before we embark on a detailed and systematic analysis of the model defined by Eq. 1, we provide an overview over the results that can be directly used for the quantitative analysis of experimental data. In a given environment (growth medium), the model as given by Eq. 1 has four parameters, the growth or death rates of normal cells and persisters and the two switching rates. While the growth or death rates can be measured directly, the switching rates are not directly observable, because switching occurs in individual cells and is also relatively rare. One way to obtain the switching rates is from the fraction of persisters in a population of cells. In particular, in a constant growth environment (medium without antibiotics), the population will grow exponentially and after some transient period, the population structure (the relative size of the subpopulations) will reach a steady-state and the fraction of persisters in the population is constant. In this situation, the persister fraction is found to be given by 
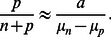
(2)


**Figure 2:**
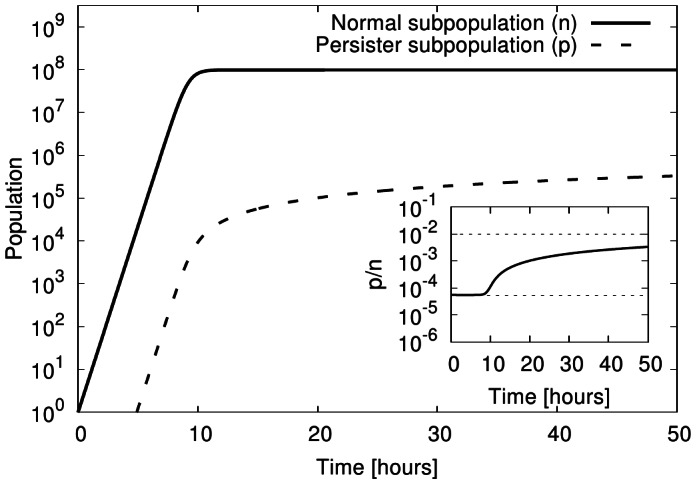
Subpopulations in exponential and stationary phase. Persister and normal subpopulation growth (with 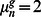 hr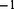, 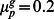 hr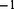, 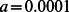 hr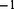, 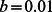 hr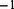) in an growth medium of carrying capacity 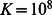 over a long period (50 hours). The inset shows the time evolution of subpopulation ratio (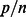) from exponentially growth phase to stationary phase. The dashed lines indicate the limits for exponential growth and no growth discussed in the text.

This result indicates that the persister fraction in an exponentially growing culture is not determined by the two-way switching between the two phenotypes. Rather, the balance is between growth and one-way switching. Normal cells, which grow more rapidly, outgrow the persisters, but they also replenish the persister subpopulation by switching to the persistent phenotype (the analogous argument can be made for stress conditions, where persisters outlast the normal cells and normal cells are re-generated by back-switching). In conditions of unstressed growth, switching of persister to the growing state has almost no impact on the persister fraction. It becomes important, however, in non-growing stationary phase cultures, which we discuss briefly below by introducing a carrying capacity of the environment into the model. In the absence of growth, the persister fraction is given by 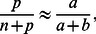
(3)


i.e. it is indeed determined by the balance of switching between the phenotypes. This observation implies that even for constant switching rates, the persister fraction increases as the cells enter stationary phase (as shown in Fig. 2). No regulatory response of the cells (to increase persister formation) is needed for this increased persister fraction. An increase of the persister subpopulation is indeed observed when cells enter stationary phase [7].

**Figure 3:**
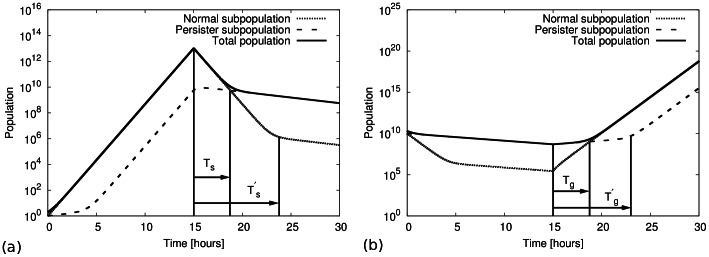
Biphasic killing kinetics. (a) Numerical integration of Eqs. (1) over a growth period of 15 hours (with 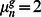 hr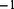, 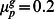 hr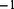) followed by a stress period (with 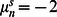 hr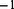, 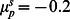 hr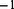) of another 15 hours. The switching rates were chosen to be 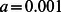 hr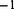 and 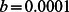 hr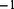. The killing curve of total population show two distinct phases, a fast-decaying phase and a slow-decaying phase. (b) Numerical integration of Eqs. (1) over a stress period of 15 hours followed by a regrowth period of another 15 hours. The regrowth curve of the total population shows two distinct phases, a slow-growing phase followed by a fast-growing phase. The parameters are the same as in (a).

A second way to determine the switching rates is via the kinetics of killing and re-growth upon addition or removal of an antibiotic. As shown in Figs. 3(a) and (b), the total population typically displays a biphasic behaviour under these conditions: Upon addition of an antibiotic, a phase of rapid decay of these population is followed by a phase of slower decay. The two phases of the double-exponential decay can be interpreted as the killing of normal cells and persisters, respectively. The time after after which killing is slowed down (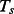) is related to the population structure at the time of addition of the antibiotic and provides another estimate of the switching rate 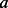 via 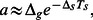
(4) where 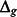 and 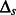 are the differences in growth and death rates, respectively, between the normal cells and the persisters. Likewise, upon removal of an antibiotic, the re-growth of the population is delayed by a time 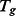 for which a similar relation is obtained, see Eq. (24).

In the following sections we will derive these expressions through a systematic analysis of the equations for phenotype switching, Eq. (1), under conditions of a constant environment, in reaction to an environmental shift and in periodically switching environments. In addition to the quantities summarized here, we will also obtain expressions for other quantities such as the growth rate and address the existence of optimal switching rates that maximize growth in periodic environments.

### Dynamics in constant environment

#### Exponential growth

In a constant environment, i.e. for fixed growth or death rates, the overall population grows or decays exponentially at long times and the relative size of the subpopulation becomes stationary. To study the fractions of the two subpopulations in the population, it is instructive to consider the time evolution of the subpopulation ratio 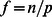, which is given by 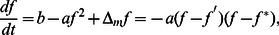
(5) where 
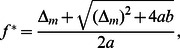


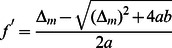
(6) and 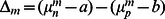. 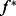 and 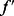 are fixed points of Eq. (5): 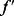 is an unstable fixed point (which moreover can be negative) and 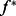 is a stable fixed point and always positive. Thus, the steady state population ratio is given by 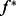.

In conditions of unstressed growth, 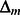 is positive. When the switching rates are small compared to the growth rates, as it is typically the case (Table 1), the steady state ratio can be approximated by 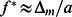 or 
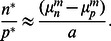
(7)


**Table 1:** Rates for switching between the normal and persister phenotype.

organism	growth rate 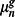 [hr 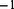 ]	growth rate 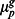 [hr 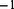 ]	Switching rate 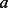 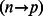 [hr 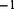 ]	switching rate 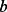 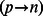 [hr 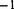 ]	reference
*E. coli*	2	0–0.2	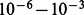	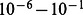	[8]
S. aureus	1.24	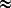 0	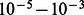	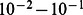	[25]

The last equation has a simple, but instructive interpretation: the steady state population structure with a certain ratio of normal and persister cells in conditions of unstressed growth is determined by a balance of two processes: The fast-growing normal cells outgrow the slow-growing persisters (with 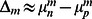), but they also replenish the persister population via switching to the persistent state (with rate 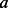). We linearize Eq. (5) around the fixed point 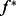 to determine the time scale in which the steady state is approached, 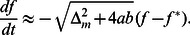
(8)


This equation shows that the subpopulation ratio 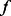 approaches the steady state 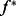 with rate 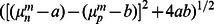, which, for small switching rates 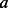 and 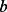, is approximately equal to the growth rate difference between the two subpopulations.

Likewise, the same approximation applied to stress condition (with 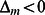), leads to the steady state 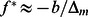 or 
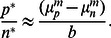
(9)


In this case, the steady state population structure is determined by the balance of persisters outlasting the normal population (with 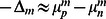) and reproducing it through phenotype switching (with rate 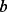).

The existence of a finite steady state in the subpopulation ratio indicates a stable coexistence of the two cells types that grow (or decay) with different rates. It is worth noting that such coexistence is an effect of phenotype switching, as normally a faster growing subpopulation will outgrow a slow-growing one, so that the subpopulation ratio will approach either zero or infinity. Here however, switching of cells between the two phenotypes that correspond to the two subpopulations can replenish the slower-growing (or faster-decaying) subpopulation and balance the outgrowth effect.

So far we have only considered the subpopulation ratio or the fractions of total population that belong to the two phenotypes. Within the model of Eqs. (1), these fractions approach a steady state, while the overall population always grows or decays exponentially on long time scales. Thus, both subpopulations grow or decay with the same average rate in the steady state, which corresponds to the effective growth rate (or decay rate) of the total population. The steady state growth rate of the total population (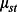) is obtained from Eqs. (1) by substituting Eq. (6) and is given by 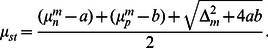
(10)


We use again an approximation of small switching rates and neglect terms of quadratic order in the switching rates (terms proportional to 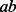). With this approximation the steady state growth rate is simplified to 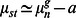
(11) in unstressed growth (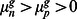) and to 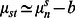
(12) under stress conditions (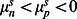).

The comparison of these two approximate expressions shows that the presence of persister cells causes a small reduction in the steady state growth rate under unstressed conditions (of order 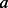), but leads to a significant reduction in the steady state death rate of the total population under stress conditions as compared to a population without any persister cells. Therefore one can expect the presence of persister cells to be beneficial provided that stress conditions do regularly occur.

In the following we will discuss the time evolution of the two subpopulations in more detail. We start by considering a constant environment. To solve the time-dependence of the coupled equations in Eqs. (1), we make once more use of the approximation for small switching rates (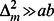).

If the constant environment is one of unstressed growth (with 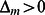), the fixed points can be approximated by 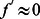 and 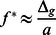. The differential equation for the subpopulation ratio (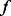) is thereby reduced to a logistic equation, 
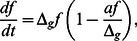
(13) where 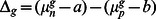. Its solution has the following form: 
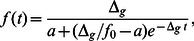
(14) where 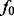 is the initial ratio of normal to persister cells.

For 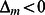, i.e. in stress conditions, a similar differential equation like Eq. (5) is obtained for the time evolution of ratio of the persister subpopulation to the normal subpopulation, 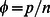. The differential equation of 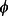 has two fixed points 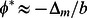 and 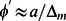, of which 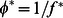 is stable. In this case, using the approximation 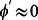, we obtain 
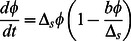
(15) with 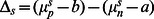. With an initial ratio 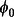, the solution has the form 
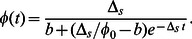
(16)


For large times, these expressions approach the steady state results derived previously and given in Eq. (7) and Eq. (9), respectively. As a consequence, under unstressed growth conditions, the effective growth rate of the persister subpopulation approaches the growth rate of the normal subpopulation in a logistic fashion and vice versa.

The functional form of 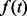 and 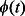 derived here will be used as the basis for our further analysis. Because of the symmetry between the two cases, we will calculate quantities in only one condition (growth or stress). The results for the other condition are obtained by simultaneously exchanging symbols and indices according to the rules 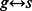, 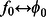, 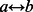, and 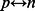.

#### Transition to stationary phase

The balance between phenotype switching and outgrowing of one subpopulation by the other that we have discussed above is intricately linked to the exponential growth or decay of the total population. While exponential growth phase is the main focus of our study, we want to briefly address the case where the population reaches a stationary phase due to a finite carrying capacity (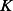) of the growth environment, a typical situation in both natural habitats and in the test tube. To this end, we modify the growth terms in Eq. (1) by multiplying them with 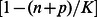. We consider the case of overall growth of the population, which is then described by 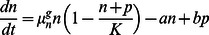


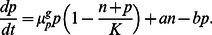
(17)


Fig. 2 shows the time evolution of the two subpopulations for an unstressed growth environment (i.e., with positive growth rates). During exponential growth phase, the total population increases exponentially with a stationary population structure as discussed above. The persister fraction is given by Eq. (7), i.e. determined by the balance of growth and persister formation. At long times, however, the population size reaches the steady-state value 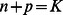. During the transition into stationary phase, the persister fraction increases. In the long-time limit, the growth terms are zero. The ratio between persisters and normal cells is then determined not by a balance of one-way phenotype switching and growth, but by a balance between switching in both directions, given by 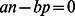. As a consequence, the ratio 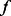 is given by 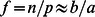. Using the parameters from Table 1, this indicates that the persister level is several orders of magnitude larger in stationary phase than than the corresponding value obtained for exponential growth, Eq. (7). This result is consistent with the observation that typically the fraction of persisters in the population is larger in stationary phase than in exponential growth phase (e.g., a 100-fold effect in *E. coli*) [7]. It is worth noting that the increase in persister level is not due to increased formation of persisters (which could for example be induced by stress responses), but by the slow-down of growth that effectively changes which processes dominate the population structure. The two limiting cases, balance between growth and one-way switching and balance of both way switching are indicated by the dashed lines in the inset of Fig. 2. Note that the approach the the long-time limit is rather slow due to the small switching rates.

### Response to environment shift

#### Characteristic time scales of the population dynamics

Next we turn to the dynamics after an environmental shift. Experimentally, one typically considers two situations [4], [8], [25]: (i) a population that has been growing under unstressed conditions for a sufficiently long time is exposed to an antibiotic or (ii) a population that has been exposed to an antibiotic for some time is shifted back to a medium without the antibiotic. In both cases, one typically observes a biphasic dynamics. For instance, a population exposed to an antibiotic typically shows biphasic decay.

Such kinetics is obtained as a consequence of the coexistence of the two phenotypes and the time at which the global decay rate changes provides an easily observable signature of phenotype switching that allows to infer its microscopic parameters.

Fig. 3(a) shows a numerical example of such dynamics: Here Eqs. (1) have been integrated to reach a steady population ratio under growth conditions with a small persister fraction. At time 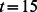 hours, the parameters were changed to those for stress condition. After the shift to stress conditions (by the addition of an antibiotic), the total population displays the biphasic decay behavior. In the fast-decaying phase, the decay of the total population is dominated by the death of normal cells, while in the second, slower-decaying phase, the total population consists predominantly of persister cells and the decay rate is governed by the death rate of the persisters. The transition between the two different phases occurs when both subpopulation becomes equal in size, i.e. at a time 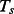 for which 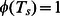. Therefore, the transition time (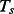) from the fast-decay phase to the slow-decay phase after the shift to stress conditions or to antibiotic-containing medium is given by 
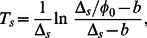
(18) where 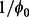 is the initial ratio of normal cells to persister cells at the time antibiotic treatment. During the growth phase, the normal cells make up the majority of the population and persister cells account for only a small fraction of the total population, which means 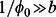. In the limit 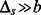, i.e. if the growth rate difference is larger compared to switching rate, the exit time can be approximated by 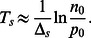
(19)


The expression for 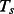 shows that the population will exit sooner from the fast-decaying phase if it has a large ratio of persister cells initially. It shows that the longer survival or persistence of the bacterial populations in antibiotic treatment depends on the fraction of persister cells that the population has formed beforehand as its survival strategy against unpredictable bad conditions.

It is worth mentioning that the time 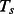, which characterizes the transition between the two phases of the decay of the total population, is not the characteristic time for reaching the new steady-state population ratio. The latter occurs later and is characterized by a time *T'_s_* that can be determined as the inflection point of the time-dependent decay rate of the normal subpopulation (calculated below), which leads to 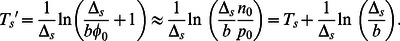
(20)


The last expression here shows directly that equilibration of the population structure occurs later than the transition in the growth rate. The delay between the two time scales is determined by a balance between the two effects that dominate the population structure under stress conditions [as in Eq. (9)], persisters taking over the population by outlasting the normal cells and switching of persisters to the normal state.

The re-growth of a population after the removal of the antibiotic is also biphasic with an initial slow-growth phase followed by a phase of rapid growth (Fig. 3(b)). The transition between the two phases can be analyzed in the same way. The transition time from the slow-growing phase to fast growing phase is given by 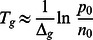
(21) and depends on the initial persister subpopulation. Therefore, a larger persister fraction under stress conditions (e.g. due to longer exposure to the antibiotic) results in a delay in resuming the maximum growth rate after the shift to conditions of unstressed growth. As above, the steady state population ratio is reached at the later time *T'_g_*, given by 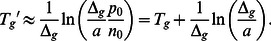
(22)


In both types of experiments, both times scales can be determined experimentally, but 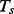 and 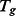 are much more easily accessible than *T'_s_* and *T'_g_*, as they only require measurements of the total population size, e.g. by colony counting, while measuring *T'_s_* or *T'_g_* requires to determine the time-dependent persister fraction. We note that the transition time 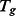 or 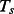 are closely related to the "delay times" defined in Ref. [22]. In fact these delay times are obtained from 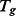 or 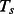 by further approximating 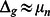 and 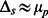. The underlying picture of Ref. [22] is that after an environmental shift, the population grows (or decays) exponentially with a new growth rate after a delay during which the population structure adjusts to the new environment. In contrast, our analysis indicates that the new steady state of the population structure is reached later than the macroscopically observable delay or transition time.

If the subpopulation ratio has reached the steady state before the shift from one environmental condition to the other, the expressions for the time scales 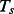 and 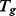 can be further simplified using Eqs. (19) and (21). As a result the transition times of total population growth or decay can be expressed in terms of the switching rates as 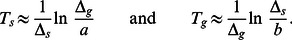
(23)


With the exception of the switching rates 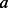 and 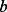, all quantities entering these equations are directly accessible though the time-dependence of the total population size (as shown in Figs. 3(a) and 3)(b) and discussed below in more detail). Thus, the phenotype switching rates can be determined from time courses of the total population size in a set of two shift experiments: (i) a sufficiently long period of of unstressed growth long followed by stress (addition of the antibiotic) and (ii) a sufficiently long stress period followed by a growth period (via shift to medium without the antibiotic). Then, the switching rates 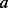 and 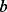 can be calculated from the parameters of the growth (or decay) curves by inverting the two equations above, 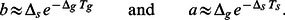
(24)


#### Time-dependent growth rates

The numerical integration of the population dynamics as plotted in Figs. 3(b) show that the growth of normal subpopulation under unstressed growth conditions is exponential with growth rate approximately given by 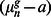, as obtained from our approximation for small switching rates above. By contrast, the growth of the persister subpopulation is biphasic and can be characterized by a time-dependent effective growth rate 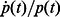. Note that this effective growth rate describes the overall growth of the persister subpopulation and includes the effects of persister proliferation and of phenotype switching.

Alternatively, it can be characterized by the average of that effective growth rate up to time 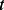, 
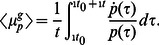
(25)


The latter quantity has the disadvantage to depend on a somewhat arbitrary initial time 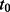, but can easily be determined experimentally from the overall increase of the persister subpopulation. The growth rate can be calculated from Eqs. (1) by substituting 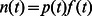, where 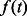 is the time-dependent subpopulation ratio that we have already calculated in Eq. (14). Therefore, the average growth rate of the normal (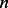) and persister (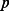) subpopulation over a growth period 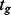 is 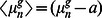


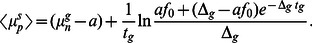
(26)


Likewise, the average growth rate for the normal (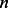) and persister (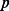) subpopulation over a stress period 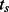 is given by 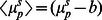


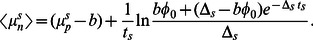
(27)


Note that in both cases the effective growth rates growth rate of both subpopulation approach the same value for large times 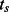 or 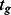.

#### Growth of the total population

Explicit expression for the effective growth rate and the time evolution of the total population can also be computed using the the results of analytical approach. The time evolution equation of total population 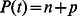 under unstressed growth conditions can be expressed in terms of subpopulation ratio 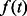 as 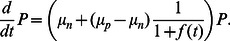
(28)


The time dependent average growth rate of the total population in a growth period 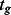 is given by 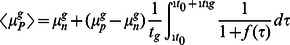


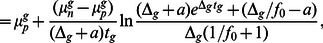
(29) where the last expression has been obtained by substituting the explicit functional form of 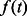.

Similarly, the average growth rate of the total population during stress conditions is given by 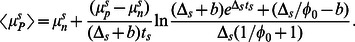
(30)


The above expression can be further simplified using again an approximation for small switching rates (compared to 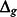 and 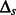). As a consequence 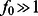, 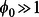. Within this approximation, the total population follows a double exponential dynamics both during unstressed growth, 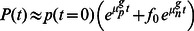
(31) and under stress, 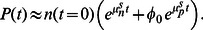
(32)


The dynamics of the total population is accessible to direct experimental observations. These expressions can therefore be used for the quantitative analysis of experimental killing curves or regrowth experiments. By fitting such data with these expressions, the growth (or death) rates and the initial fractions of the subpopulation can be obtained [25].

### Dynamics in periodically switching environment

Finally, we consider an environment that switches periodically between growth and stress conditions. The duration of the conditions are denoted by 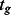 and 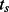 (for a test of our approximation in this case, see Text S1 and Fig. S1) To address the evolutionary consequence of phenotype switching in varying environmental conditions, we calculate the average growth of the population over one environmental cycle. The average growth rate of the subpopulation over one environmental cycle of duration 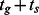 are given by 
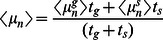
(33)

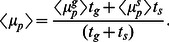
(34)


In general, these expressions depend on the initial subpopulation ratio during growth and stress. Here we are most interested in the long-time behaviour, where the population sizes are periodic. In that case, the effective growth rates of both subpopulations are equal. Expressions for the growth rates for several cases are given in Text S1.

Here we focus on the case, where both phases of the environmental cycle are sufficiently long such that a steady state of the population structure is reached in each condition. In that case, the average growth rate is given by 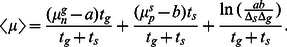
(35)


**Figure 4:**
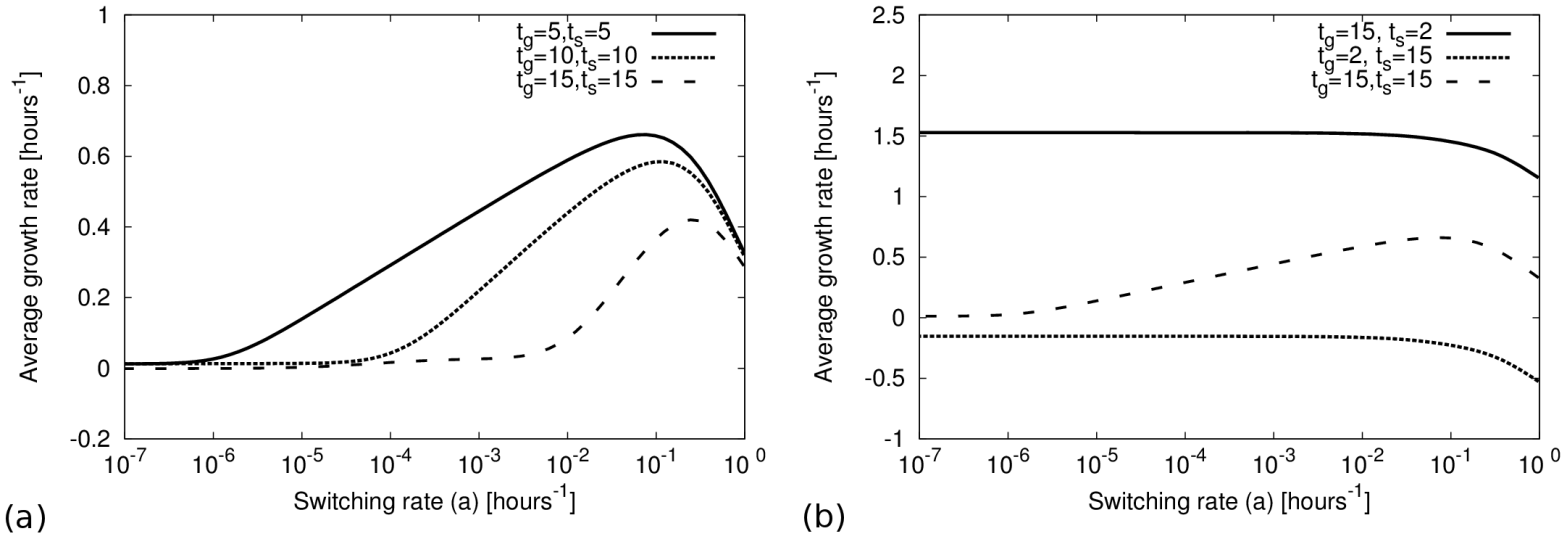
Optimal switching rate. (a) The average growth rate over an environmental cycle is plotted as a function of the phenotype switching rate (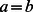) for different environmental durations (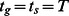). The figure shows the existence of an optimal switching rate for a given cycle duration 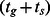. (b) Existence of an optimal switching rate: Average growth rate as function of the switching rate (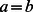). An optimal switching rate is seen for slowly varying environment, but not if one environmental duration is short. The growth rates are the same as in Fig. 3.

It is tempting to speculate that the population might adapt under such repeated environmental conditions to maximize the average growth rate. The growth and killing rates of the normal and persister cells are environment-dependent but the switching rates can be tuned to maximize the average growth rate for a given environmental periodicity [23], [26], as shown in Fig. 4(a). (In a more realistic description, the environment may be varying stochastically rather than in a strictly periodic fashion. Here we take this duration in our period environment as representative of the typical duration of a cycle in a stochastically varying environment, but note that in the stochastic case, extremes of the duration, e.g. very short growth persiods or very long stress periods, may have additional impact.) The optimal values for 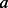 and 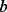 can be calculated by maximizing above expression, which leads to 
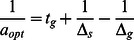


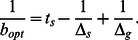
(36)


The dominant term in the expression for the optimal switching rates (36) are the environmental durations 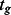 and 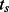. The correction terms are in principle dependent on the switching rates. As these expressions are valid for long environmental durations, and thus small switching rates, this dependence can be neglected. So the correction terms in this expression should therefore be taken at zero switching rates. Alternatively, Eqs. (36) can be interpreted as implicit equations and solved for the switching rates. These expressions in Eq. (36) are similar to the expression given by Kussel et al. [23], which indicates the consistency between different theoretical approaches. Similar results on the existence of such optimal switching have also been obtained in a number of other previous studies [22], [26], [27].

It is worth noting that a maximum of the growth rate and thus optimal switching rates are found only when the environment changes very slowly, as shown in Fig. 4(b). This is different from the behaviour described in previous studies [22], [23], which focused on the limit of long environmental durations. Approximations for this limit cannot predict the growth rates for cases, where one of the environmental durations is short (e.g., for very brief exposures to antibiotics). Within our approximation this case can be addressed. We find that if one of the environmental duration is short, the growth rate decreases with the increasing phenotype switching rate, which suggests that phenotype switching is unprofitable under such conditions (see the expressions for cases 2 and 3 given in Text S1).

In our analysis, we have focused on exponentially growing cells, but as mentioned already, in natural environments, growth is usually limited by the carrying capacity of the environment, so we want to briefly mention how the dynamics is affected by such carrying capacity. We have shown above that, under constant conditions promoting growth, the total population will eventually reach the carrying capacity and that in this steady state the subpopulation ratio is determined by the phenotype switching rates, 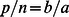 (as shown in Fig. 2). In stress conditions, the dynamics is unaffected by the environmental carrying capacity. The dynamics in periodically switching environments depends on whether the average growth rate (in the absence of a carrying capacity) is positive or negative. In the case of net decay, our analysis remains valid, as the carrying capacity is irrelevant. But if there is net growth per environmental cycle, the population will eventually grow to the carrying capacity during a growth period. From then on, the population will oscillate between decaying away from the maximal population size during the stress period and growing back to it in the growth period. The long term growth rate is zero in this case.

## Concluding Remarks

One way bacterial population cope with environmental stresses is by setting aside a small fraction of the total population, the persister cells, in a slow-growing, but stress-tolerant phenotypic state. These persisters provide a pool of cells from which the population can recover via a phenotypic switch to the normal growth state after the environmental conditions have improved. Here we have analyzed a simple mathematical model to understand the dynamics of phenotype switching. Typically, the fitness cost associated with the switching of few normal cells to the persister phenotype under growth-permissible conditions is small compared to the fitness benefit of the presence of persister cells under stress conditions. We have used an approximation valid for small switching rates that allows us to obtain explicit analytical expression for many quantities that are directly accessible to experiments. Within this approximation, the population dynamics is mapped to a logistic equation for the ratio of the population fractions corresponding to the two phenotypes. For constant environmental conditions, stable coexistence of the two subpopulations that grow with different growth rates is achieved by a balance between fast-growing cells outgrowing the slow-growing ones and phenotype switching, by which the slow-growing subpopulation is replenished.

We have then considered shifts between environmental conditions as well as periodically switching environments. Specifically, we have identified several characteristic time scales for changes in the overall population growth or decay and for the approach to a constant ratio between the two subpopulations. Simple analytical expressions for these time scales provide a window into the phenotype switching process and more specifically allow to determine the switching rates from population-scale shift experiments [25], which are typically governed by double-exponential population growth or decay. Finally, we determined the average growth rates for a periodically switching environment. If growth and stress periods have long durations, the phenotype switching rates can be tuned for optimal growth of the total population.

The results derived are based on the assumption that phenotype switching is a stochastic process, independent of the environment. This assumption may not always be valid, as in some cases, persistence may also involve an adaptive response to the stress conditions. One case, where this has been demonstrated is persistence of *E. coli* cells upon treatment with the antibiotic Ciprofloxacin, where persistence is actively induced via the SOS response [9], [10]. Analysis of such cases with our model would lead to condition-dependent apparent switching rates. In such cases, the model may be used as a 'null model' to identify deviations from the simple dynamics discussed here.

Finally, we want to emphasize that the analysis we have developed here, can also be applied to other cases of 'growth bistability', i.e. cases of phenotypic heterogeneity, where genetically identical subpopulations grow with different growth rates. One interesting case is bacterial competence, a program for genetic transformation (quasi-sexual exchange of genetic material), which is typically activated in only a subpoulation [3]. In this case, it has been proposed that phenotypic heterogeneity provides a evolutionary advantage in a homogeneous environment [28]. Recent studies of growth effects on various genetic circuits suggest that growth bistability may be a rather generic consequence of the coupling of gene expression and cell growth [17], [20], [29], [30].

## Supporting Information

Figure S1
**Comparison of the approximation for small switching rates with the exact numerical result.** Steady-state ratio of the normal to the persister subpopulation at the end of an environmental cycle (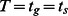). The parameters are the same as in Fig. 3.(TIF)

Text S1 **Average growth rate in periodic environmental conditions, steady state ratio of subpopulations.**
(PDF)
