# Supplementary material for: Population Dynamics of Bacterial Persistence
Source: PLoS One. 2013 May 13;8(5):e62814. doi: 10.1371/journal.pone.0062814 (PMC3652822; doi:10.1371/journal.pone.0062814)
Supplement: Text S1 — Average growth rate in periodic environmental conditions, steady state ratio of subpopulations. (PDF) [file pone.0062814.s002.pdf]

Supporting Text

Average growth rate in periodic environmental conditions

In this appendix, we give expression for the average growth rate of the total population for several different cases beyond the case of slow environmental variation discussed in the main text.

Case 1: $t_g > T'_g$ and $t_s > T'_s$. For slow variation of the environment, i.e. for long durations of the growth and stress periods, the average growth rate is given by

$$\langle\mu\rangle = \frac{(\mu_n^g - a)t_g}{t_g + t_s} + \frac{(\mu_p^s - b)t_s}{t_g + t_s} + \frac{\ln(\frac{ab}{\Delta_s \Delta_g})}{t_g + t_s}$$

Case 2: $t_g > T'_g$ and t_s is very small. For short duration of the stress period and long duration of the growth period, the average growth rate is given by

$$\langle\mu\rangle = \frac{(\mu_n^g - a)t_g}{t_g + t_s} + \frac{(\mu_p^s - b)t_s}{t_g + t_s} + \frac{\ln\left(\frac{ab + (\Delta_s \Delta_g - ab)e^{-\Delta_s t_s}}{\Delta_s \Delta_g}\right)}{t_g + t_s}.$$

The average growth rate decreases as the switching rate increases as shown in Fig. 4(b). The maximal growth rate for infinitesimally small switching rates ($a \to 0$ and $b \to 0$) is given by

$$\langle\mu\rangle_{max} = \frac{(\mu_n^g - a)t_g}{t_g + t_s} + \frac{(\mu_n^s - a)t_s}{t_g + t_s}.$$

Case 3: $t_s > T'_s$ and t_g is very small. Likewise, for short duration of the growth period and long duration of the stress period, the average growth rate is given by

$$\langle\mu\rangle = \frac{(\mu_p^s - b)t_s}{t_g + t_s} + \frac{(\mu_n^s - a)t_g}{t_g + t_s} + \frac{\ln\left(\frac{ab + (\Delta_s \Delta_g - ab)e^{-\Delta_g t_g}}{\Delta_s \Delta_g}\right)}{t_g + t_s}.$$

Again, the average growth rate decreases as the switching rate increases as shown in Fig. 4(b). The maximum growth rate for small switching rates ($a \to 0$ and $b \to 0$) is given by

$$\langle\mu\rangle_{max} = \frac{(\mu_p^g - b)t_g}{t_g + t_s} + \frac{(\mu_p^s - b)t_s}{t_g + t_s}.$$

Steady state ratio of subpopulations

In this paper, we make extensive use of approximations based on the observation that the switching rates a and b are typically very small (Table.1). As a numerical test of this approximation, we consider the subpopulation ratio, which becomes constant over long times of constant growth or decay conditions. In periodically varying environment the ratio of the subpopulations at

end of an environmental duration is determined by the environmental periodicity (the durations of the two phases, t_g and t_s). The exact steady state ratio of the subpopulations can be determined numerically by integrating the dynamical equations Eqs. (1) over a long time under a given environmental condition. Here, we compare the value of the subpopulation ratio $\phi(t_g, t_s)$ at the end of a stress phase as obtained from our analytical approximation with the corresponding numerical result. The analytical result is obtained by iterating the following recursion

$$\begin{aligned} f(t_g, t_s) &= \frac{\Delta_g}{a + (\Delta_s \phi(t_g, t_s) - a)\ e^{-\Delta_g t_g}} \\ \phi(t_g, t_s) &= \frac{\Delta_s}{b + (\Delta_g f(t_g, t_s) - b)\ e^{-\Delta_s t_s}}. \end{aligned}$$

The comparison between the numerical and analytical results are plotted in Fig. S1, which shows good agreement between the two methods.
